# Low Skeletal Muscle Mass Impairs Quality of Life in Nasopharyngeal Carcinoma Patients Treated With Concurrent Chemoradiotherapy

**DOI:** 10.3389/fnut.2019.00195

**Published:** 2020-01-15

**Authors:** Xin Hua, Jun-Fang Liao, Shan Liu, Jun Zhang, Han-Ying Huang, Wen Wen, Zhi-Qing Long, Wen-Wen Zhang, Ling Guo, Huan-Xin Lin

**Affiliations:** ^1^State Key Laboratory of Oncology in South China, Guangdong Key Laboratory of Nasopharyngeal Carcinoma Diagnosis and Therapy, Sun Yat-sen University Cancer Center, Collaborative Innovation Center for Cancer Medicine, Guangzhou, China; ^2^Department of Medical Oncology, Sun Yat-sen University Cancer Center, Guangzhou, China; ^3^Department of Radiation Oncology, National Cancer Center/National Clinical Research Center for Cancer/Cancer Hospital & Shenzhen Hospital, Chinese Academy of Medical Sciences and Peking Union Medical College, Shenzhen, China; ^4^Department of Radiation Oncology, Sichuan Cancer Center, School of Medicine, Sichuan Cancer Hospital & Institute, University of Electronic Science and Technology of China, Chengdu, China; ^5^Department of Radiotherapy, Sun Yat-sen University Cancer Center, Guangzhou, China; ^6^Department of Nasopharyngeal Carcinoma, Sun Yat-sen University Cancer Center, Guangzhou, China

**Keywords:** nasopharyngeal carcinoma, low skeletal muscle mass, pain, quality of life, concurrent chemoradiotherapy

## Abstract

**Background:** Nasopharyngeal carcinoma (NPC) patients receiving concurrent chemoradiotherapy (CCRT) frequently develop low skeletal muscle mass (SMM), but, little is known about the impacts of low SMM on health-related quality of life (QOL).

**Methods:** We retrospectively assessed 56 patients with locoregionally advanced NPC enrolled in a prospective trial. Low SMM was determined on routine computed tomography simulation (CT-sim) scans taken before radiotherapy, at the third cervical (C3) vertebral level with validated sex-specific cutoffs. QOL was assessed using the World Health Organization Quality of Life Questionnaire-100 at baseline and after 3 weeks. Pain was scored every 24 h using a numerical rating scale (NRS). Characteristics related to low SMM were identified by logistic regression. The chi-square test was used to examine the association of low SMM with QOL and pain.

**Results:** Of the 56 participants (mean age 44.20 ± 10.93 years), over half (60.71%) developed low SMM. Patients with low SMM were more likely to be older (*P* = 0.035), male (*P* = 0.066), have a lower body-mass index (BMI; *P* = 0.091), and have a higher pain score (*P* = 0.001). Older age (hazard ratio [HR] = 1.788, *P* = 0.016), being male (HR = 3.145, *P* = 0.010), lower BMI (HR = 0.761, *P* = 0.033), and lower prognostic nutritional index (HR = 0.186, *P* = 0.034) were associated with higher risk of low SMM. Low SMM was associated with poorer baseline QOL scores (*P* = 0.072), especially in the physical domain (*P* = 0.002) and its three facets: pain (*P* = 0.003), energy (*P* = 0.021), and sleep (*P* = 0.007). Low SMM was also associated with significantly worse QOL scores (*P* = 0.006) at 3 weeks, especially in the physical (*P* = 0.002), psychological (*P* = 0.046), independence (*P* = 0.003), social domains (*P* = 0.023), and in general health condition (*P* = 0.043). For pain score, low SMM group had worse overall changes from baseline to week 3 (*P* = 0.011).

**Conclusions:** The incidence of low SMM, as evaluated using routine CT-sim scans, is high in patients receiving CCRT for locoregionally advanced NPC. Low SMM results in poorer QOL and higher pain scores, which underscores the requirement for nutritional and functional interventions to address low SMM early in the treatment course.

## Introduction

Nasopharyngeal carcinoma (NPC) is an epithelial carcinoma with distinct pathophysiological characteristics in contrast with other head and neck cancer (1, 2). The mainstay of treatment of locoregionally advanced NPC is concurrent chemoradiotherapy (CCRT) (3). The most common CCRT-related toxicities include mucositis, dermatitis, nausea, poor appetite, and dysphagia (4). Intensity-modulated radiotherapy (IMRT), which is currently the most common photon-based radiotherapy technique used in clinical practice, does to some degree reduce treatment-related toxicity and improve patient quality of life (QOL) (5). However, adding chemotherapy to IMRT inevitably increases the treatment-related toxicities and deteriorates patient QOL, thereby impairing treatment compliance and even having life-threatening consequences. Almost all patients who undergo CCRT develop some toxicities, which increases the risk of decreased physical activity, reduced appetite, and metabolic disorders (6).

Sarcopenia was defined as generalized and progressive loss of skeletal muscle strength (SMS) and skeletal muscle mass (SMM) in various clinical settings (7). For many cancer patients with sarcopenia, especially for those treated by sequential chemotherapy and radiotherapy, may experience more serious chemotherapy dose-limiting toxicity, poorer treatment tolerance and worse survival (8, 9) which also occurs in locoregionally advanced head and neck cancers (10, 11). While many researchers have investigated the effects of sarcopenia on prognosis in head and neck cancers, the association with patient-reported QOL remains unclear (8, 10–12). Our previous study found that QOL can affect patient response to treatment, as well as long-term survival (13, 14). Importantly, the deterioration in QOL caused by sarcopenia often appears gradually, as the cumulative dose of radio- and chemotherapy increases. Hence, clinicians may only notice the profound deterioration in QOL during the later stages of treatment, when it is too late to reverse by intervention. We should determine the associations between sarcopenia and patient QOL, to facilitate earlier intervention.

Here, we retrospectively analyzed data from a prospective clinical trial which evaluated the efficacy of oxycodone in reducing treatment-related mucositis pain of CCRT for NPC. We used loss of SMM as an indicator of sarcopenia to determine the association between sarcopenia before treatment and the QOL at different timepoints. And relationship between low SMM and patient characteristics and therapeutic response were also assessed in detail.

## Methods

### Study Population

This study enrolled 56 NPC patients who received CCRT between 19th May 2015 and 23rd January 2018 at Sun Yat-sen University Cancer Center, Guangzhou, China, as part of a prospective clinical trial. The details of this trial have been described by Hua et al. (13). In brief, all patients received triweekly cisplatin (100 mg/m^2^)-based CCRT concurrently with IMRT following the guidelines of our institute (15), and reported moderate or severe pain, as indicated by numerical rating scale [NRS] pain scores of 4–10. This study was approved by the Institutional Review Board of Sun Yat-sen University Cancer Center. Written informed consent was obtained from all patients.

### Routine Assessments

We collected pretreatment laboratory data from within 1 week of CCRT as well as clinicopathological data from patient medical records. Plasma Epstein-Barr virus DNA levels (copies/mL) were measured using a real-time quantitative polymerase chain reaction assay, and its cutoff value classified as previously described (16, 17). Representative hematological biomarkers, such as the neutrophil to lymphocyte ratio (NLR), platelet to lymphocyte ratio (PLR), monocyte to lymphocyte ratio (MLR), and prognostic nutritional index (PNI) were assessed, and their cutoffs classified as previously described (18–20). All patients were observed from fraction 15th to 30th of radiotherapy for 3 weeks. And pain was assessed daily using the NRS. Nasopharyngoscopic examinations were conducted weekly and magnetic resonance imaging (MRI) was conducted at 30th radiotherapy fraction to compare the therapeutic effects of CCRT. Complete response (CR) was defined as complete regression of observable nasopharyngeal primary tumors by imaging methods above.

### SMM Assessment

**Figure 1:**
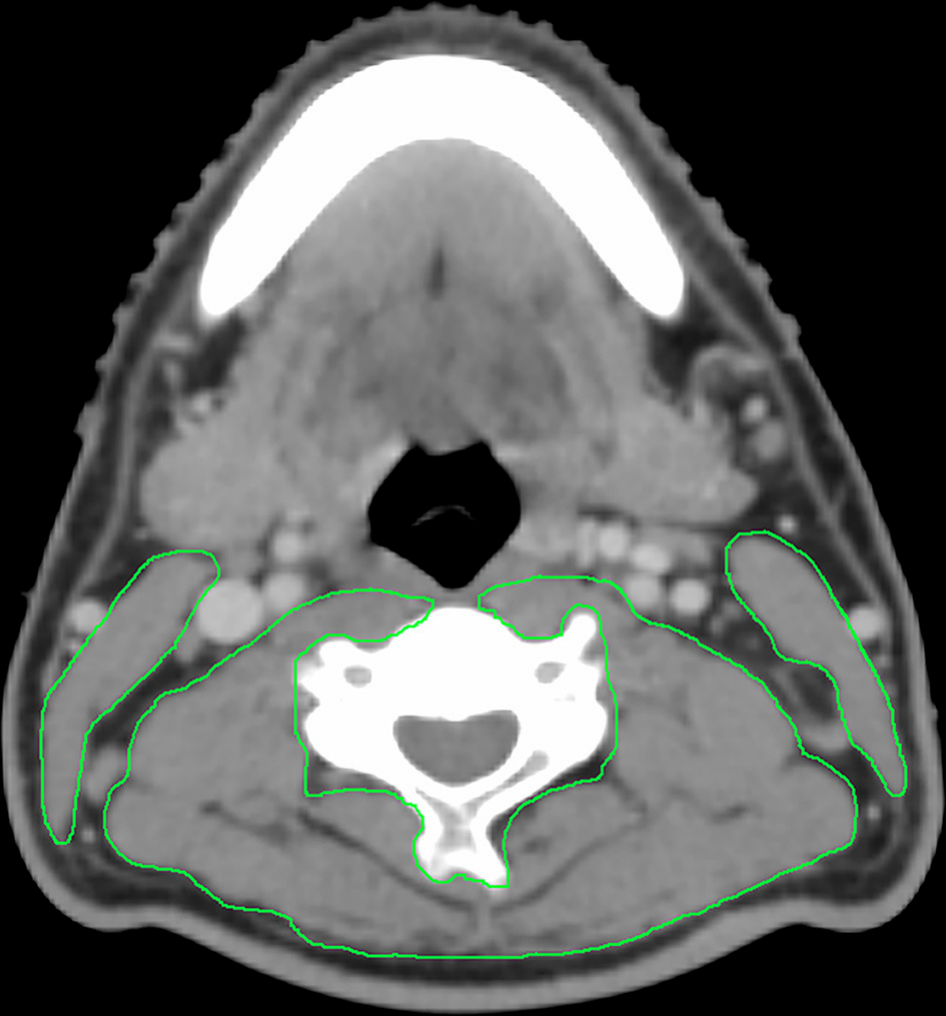
Example contour of the sternocleidomastoid and paravertebral muscles on an axial slice at the C3 vertebral body.

On routine computed tomography simulation (CT-sim) images before radiotherapy, we measured the cross-sectional area (CSA) of the sternocleidomastoid and paravertebral muscles on an axial slice at the level of the third cervical (C3) vertebrae, the CT Hounsfield unit thresholds was −29 to +150 for skeletal muscle (Figure 1), as previously described (8, 21). Monaco TPS software (version 5.1, Elekta CMS, Maryland Heights, MO, USA) was used for image evaluation, registration, and mapping at our center for radiotherapy planning. The contours of the muscles were hand-drawn by a senior radiotherapy specialist (LG) and reviewed by another senior specialist (HXL). The CSA of skeletal muscles at the third lumbar (L3) vertebral level was calculated using the formula described by Jung et al. (21): CSA at L3 (cm^2^) = 81.059 + 0.874 × CSA at C3 (cm^2^) + 0.956 × Weight (kg) – 28.127 × Sex (value = 1 for female and 2 for male) – 0.257 × Age (y). The skeletal muscle index (SMI) which represented generalized SMM was calculated as follows: SMI (cm^2^/m^2^) = CSA at L3 (cm^2^)/height^2^ (m^2^). Low SMM was defined (8) as an SMI of <41 cm^2^/m^2^, regardless of body-mass index (BMI) in the case of female patients, and as an SMI of <43 cm^2^/m^2^ in male patients with BMI ≤ 25 or an SMI of <53 cm^2^/m^2^ in male patients with BMI >25.

### QOL Assessment

QOL was assessed at baseline and after 3 weeks using the World Health Organization QOL Questionnaire-100 (WHO QoL-100) (22). The WHO QOL-100 contains 100 items with subscales assessing wellbeing across six domains: physical health, level of independence, social relations, psychological, environment, and spirituality/religion/personal beliefs. Higher scores on the WHO QOL-100 indicate better QOL. An additional question concerning appetite was assessed and analyzed separately.

### Statistical Analyses

Data were analyzed using SPSS software (v. 19.0; IBM Corp., Armonk, NY, USA). Missing data in QOL analysis were handled using mean/mode imputation. After test of normality of distribution, student's *t*-test or Mann–Whitney *U*-test were used to compare continuous variables, and the chi-squared test and Fisher's exact test to compare categorical variables. Logistic regression, adjusted for variables with *P* < 0.25 in univariate analysis, was used to identify patient characteristics independently associated with the presence of low SMM. All statistical tests were two-sided. *P* ≤ 0.05 was considered statistically significant.

## Results

### Patient Characteristics

The present observational, retrospective study evaluated data for patients (*n* = 56) who were enrolled in our previous prospective trial. Their baseline characteristics are shown in Table 1. The participants (mean age, 44.20 ± 10.93 years) were primarily male, and over half (60.71%) had low SMM. Patients in low SMM group were more likely to be above 45 years old (*P* = 0.035), male (*P* = 0.066), have a lower BMI (*P* = 0.091), and have a higher NRS pain score (*P* = 0.001) (Table 1).

**Table 1:** Demographics and clinical characteristics of patients.

**Variable**		**Low SMM**	**Normal SMM**	***P***
Total (n)		34	22	
Age (years) (*n*%)	>45	19 (55.9)	6 (27.3)	0.035^a^
	≤ 45	15 (44.1)	16 (72.7)	
Gender (*n*%)	Male	31 (91.2)	16 (72.7)	0.066^c^
	Female	3 (8.8)	6 (27.3)	
BMI (kg/m^2^) (*n*%)	<18.5	4 (11.8)	2 (9.1)	0.091^a^
	18.5–25	19 (55.9)	12 (54.5)	
	≥25	11 (32.4)	8 (33.3)	
AJCC stage (*n*%)	IV	11 (32.4)	3 (13.6)	0.196^b^
	III	20 (58.8)	18 (81.8)	
	II	3 (8.8)	1 (4.5)	
Pain (NRS score) (*n*%)	4–6	6 (17.6)	21 (95.5)	0.001^c^
	7–10	28 (82.4)	1 (4.5)	
EBV-DNA	≤ 4,000	27 (79.4)	19 (86.4)	0.507^c^
	>4,000	7 (20.6)	3 (13.6)	
NLR	≤ 2.50	22 (64.7)	14 (63.6)	0.935^b^
	>2.50	12 (35.3)	8 (36.4)	
PLR	≤ 112	13 (38.2)	6 (27.3)	0.397^b^
	>112	21 (61.8)	16 (72.7)	
MLR	≤ 0.25	13 (38.2)	6 (27.3)	0.397^b^
	>0.25	21 (61.8)	16 (72.3)	
PNI	≤ 52.0	18 (52.9)	7 (31.8)	0.120^b^
	>52.0	16 (47.1)	15 (68.2)	

*BMI, body mass index; AJCC stage, American Joint Committee on Cancer 7.0; NRS, numerical rating scale; EBV-DNA, Epstein-Barr virus DNA; NLR, neutrophil to lymphocyte ratio; PLR, platelet to lymphocyte ratio; MLR, monocyte to lymphocyte ratio; PNI, prognostic nutritional index*.

a *Student's t-test*.

b *Pearson's χ^2^-test*.

c *Mann–Whitney U-test*.

### Associations Between Low SMM and Patient Characteristics

Using univariate and multivariate logistic regressions, we found that older age (hazard ratio [HR] = 1.788, *P* = 0.016) and being male (HR = 3.145, *P* = 0.010) were associated with higher risk of low SMM, while higher BMI (HR = 0.761, *P* = 0.033) and PNI (hazard ratio = 0.186, *P* = 0.034) were associated with lower risk (Table 2).

**Table 2:** Associations between patient characteristics and low SMM.

**Characteristic**	**Univariate analysis**		**Multivariate analysis**	
	**Regression coefficient (SE)**	***P***	**Hazard ratio (95% CI)**	***P***
Age (years)	1.217 (0.590)	0.039	1.788 (1.392–25.646)	0.016
Gender	1.355 (0.771)	0.079	3.145 (2.116–4.782)	0.010
BMI	−0.152 (0.091)	0.096	0.761 (0.591–0.978)	0.033
AJCC Stage	0.510 (0.525)	0.331		
EBV-DNA	0.496 (0.752)	0.510		
NLR	−0.047 (0.570)	0.935		
PLR	−0.501 (0.595)	0.399		
MLR	−0.501 (0.595)	0.399		
PNI	−0.880 (0.572)	0.124	0.186 (0.039–0.879)	0.034

*BMI, body mass index; AJCC stage, American Joint Committee on Cancer 7.0; EBV-DNA, Epstein-Barr virus DNA; NLR, neutrophil to lymphocyte ratio; PLR, platelet to lymphocyte ratio; MLR, monocyte to lymphocyte ratio; PNI, prognostic nutritional index; SE, standard error; CI, confidence interval*.

### Associations Between Low SMM and QOL

**Figure 2:**
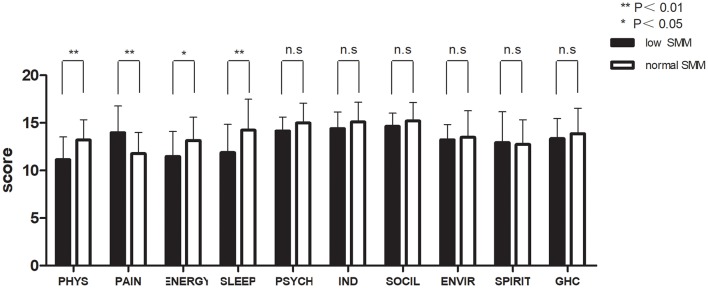
Patient-reported World Health Organization QOL Questionnaire-100 (WHO QOL-100 scores) at baseline in patients with low and normal SMM. Data are expressed as mean ± SD. PHYS, physical; PSYCH, psychological; IND, independence; SOCIL, social; ENVIR, environment; GHC, general health condition.

**Figure 3:**
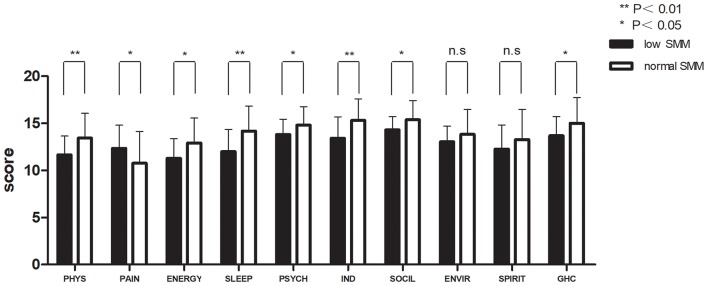
Patient-reported WHO QOL-100 scores at the end of week 3 in patients with low and normal SMM. Data are expressed as mean ± SD. PHYS, physical; PSYCH, psychological; IND, independence; SOCIL, social; ENVIR, environment; GHC, general health condition.

Figures 2, 3 and Table 3 show the mean QOL scores at baseline and after 3 weeks in both groups. At baseline, low SMM was associated with worse QOL scores (*P* = 0.072), especially in the physical health domain (*P* = 0.002), which includes the following three facets: pain and discomfort (*P* = 0.003), energy and fatigue (*P* = 0.021), and sleep and rest (*P* = 0.007). At the end of week 3, low SMM was associated with significantly worse QOL scores (*P* = 0.006) in the domains physical health (*P* = 0.002), psychological health (*P* = 0.046), level of independence (*P* = 0.003), and social relations (*P* = 0.023), and in general health condition (*P* = 0.043). There was a significant decrease in independence (low SMM group: *P* = 0.004, normal SMM group: *P* = 0.028) and pain (low SMM group: *P* = 0.001, normal SMM group: *P* = 0.001) in both groups between baseline and the end of week 3. During the same time interval, the normal SMM group alone showed an increase in the general health condition score (*P* = 0.032). The overall changes in the QOL score from baseline to the end of week 3 were comparable between the groups.

**Table 3:** Patient-reported WHO QOL-100 scores at the baseline and at the end of week 3 in patients with low and normal SMM.

**Variable**	**Score at baseline**		***P***	**Score at week3**		***P***	**Score change from baseline to week3**				***P***
	**low SMM**	**Normal SMM**		**low SMM**	**Normal SMM**		**low SMMa**	**P^a^**	**Normal SMM**	**P^a^**	
Total score	80.45 ± 7.34	84.71 ± 10.02	0.072	78.49 ± 8.27	86.02 ± 11.53	**0.006**	−1.90. ± 6.42	0.089	−0.67 ± 6.72	0.458	0.076
Physical	11.13 ± 2.39	13.20 ± 2.13	**0.002**	11.65 ± 1.99	13.44 ± 2.62	**0.005**	0.505 ± 1.90	0.126	0.41 ± 1.73	0.081	0.563
Pain	13.97 ± 2.81	11.77 ± 2.22	**0.003**	12.35 ± 2.45	10.77 ± 3.37	**0.047**	−1.57 ± 2.37	**0.001**	−1.38 ± 2.52	**0.001**	0.375
Energy	11.47 ± 2.63	13.14 ± 2.46	**0.021**	11.29 ± 2.07	12.91 ± 2.65	**0.014**	−0.17 ± 2.05	0.624	−0.20 ± 1.87	0.436	0.922
Sleep	11.88 ± 2.96	14.23 ± 3.25	**0.007**	12.00 ± 2.35	14.18 ± 2.65	**0.002**	0.11 ± 2.67	0.801	0.05 ± 2.55	0.876	0.817
Psychological	14.15 ± 1.47	14.99 ± 2.09	0.082	13.81 ± 1.61	14.80 ± 1.98	**0.046**	−0.33 ± 1.34	0.160	−0.28 ± 1.34	0.127	0.698
Independence	14.39 ± 1.75	15.10 ± 2.07	0.171	13.41 ± 2.25	15.31 ± 2.28	**0.003**	−0.95 ± 1.79	**0.004**	−0.51 ± 1.70	**0.028**	**0.010**
Social	14.63 ± 1.40	15.21 ± 1.93	0.195	14.31 ± 1.39	15.38 ± 2.03	**0.023**	−0.30 ± 1.31	0.178	−0.13 ± 1.33	0.485	0.189
Environment	13.21 ± 1.61	13.48 ± 2.79	0.648	13.04 ± 1.66	13.82 ± 2.66	0.182	−0.16 ± 1.20	0.425	0.03 ± 1.45	0.872	0.201
Spirit	12.94 ± 3.23	12.73 ± 2.59	0.795	12.27 ± 2.56	13.27 ± 3.20	0.198	−0.66 ± 2.21	0.087	−1.96 ± 2.65	0.581	0.092
General health condition	13.35 ± 2.10	13.86 ± 2.68	0.429	13.68 ± 2.04	15.00 ± 2.73	**0.043**	0.31 ± 1.98	0.355	0.64 ± 2.19	**0.032**	0.176

*WHO QOL-100: Self-administered WHO Quality of Life Questionnaire-100*.

*Results are presented as mean ± standard deviation*.

*P-value: difference between the low SMM and normal SMM groups*.

a *P-value: difference between the baseline score and the week-3 score in the low SMM and normal SMM groups. Significant results are shown in bold font*.

### NRS Pain Score

**Figure 4:**
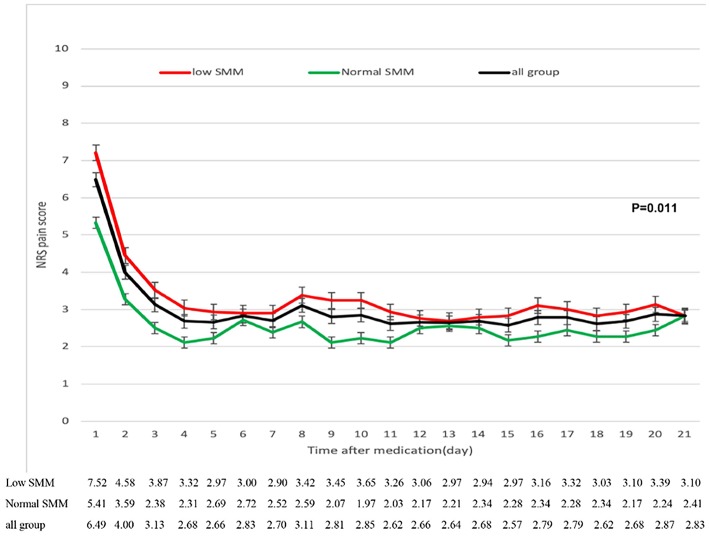
Reduction in numerical rating scale (NRS) pain scores from baseline to week 3 in patients with low and normal SMM. The error bars represent standard deviations.

In all group patients, pain was significantly reduced over the first 3 days, then effectively and steadily controlled from day 4 (Figure 4). The low SMM group reported slightly higher pain scores than the normal SMM group and the overall changes in pain score were significant (3.27 ± 0.98 vs. 2.57 ± 0.69, *P* = 0.011).

### Appetite

Patients in the normal SMM group had a significantly better appetite at both baseline (3.32 ± 0.99 vs. 2.68 ± 0.64; *P* = 0.005) and at the end of week 3 (3.22 ± 1.11 vs. 2.38 ± 0.89; *P* = 0.003) compared with those in the low SMM group.

### Response to CCRT

The mean rate of CR was 66.1% (37/56). The rate of CR was higher (in the normal SMM group [68.2% (22/34)] than in the low SMM group [64.7% (15/22)], but the *p*-value is not significant (*P* = 0.788).

## Discussion

Here, in NPC patients who received CCRT, low SMM was associated with worse patient-reported QOL both at the baseline and at the end of CCRT. We also demonstrated a significant correlation between low SMM and certain patient characteristics, including age, sex, and BMI. Additionally, we found that although patients with low SMM had a lower appetite and higher pain scores than patients without low SMM, the response to CCRT and the overall change in pain scores were comparable in the two groups.

In this study, as an indicator of sarcopenia, low SMM was indirectly measured using a low SMI at the C3 vertebral level. In most studies (12), low SMM has been determined using CT or positron-emission tomography (PET)-CT scans at the L3 vertebral level, which had been routinely performed for diagnostic purposes. These types of scan are not routinely performed for NPC patients. However, comparisons of skeletal muscle CSA at the C3 and L3 levels have demonstrated that CT scans taken at the C3 level can be used to reliably and effectively determine low SMM in patients with head and neck cancer (23). Notably, studies with large sample sizes have assessed and confirmed the efficacy of this method in European and Asian cohorts (8, 21). Every NPC patient routinely completes comprehensive pretreatment CT examinations that includes scans taken from the level of frontal sinus to the level of clavicle, which includes the C3 vertebrae. Therefore, CSA assessment at the C3 level is a reasonable and efficient method of detecting low SMM in these patients, as this method does not involve additional expense or radiation exposure to the patients.

In our study, the incidence rate of low SMM was 60.71%, which is higher than the incidence rates reported in other publications on low SMM in head and neck cancers, ranging from 6.6 to 64.6% (8, 10, 24, 25). A possible explanation for this may be that the present study enrolled patients with locoregionally advanced cancers who were receiving CCRT, whereas more patients in early stages were included in prior studies. Additionally, the treatments in previous studies consisted of radiotherapy or chemotherapy administered separately, rather than the highly intensive CCRT regimen used in our study. The high incidence of low SMM in our study cohort, combined with its negative effect on health-related outcomes, underscores the urgency to appreciate and address low SMM at an early stage of CCRT for NPC.

Multiple studies have found that QOL is an important prognostic factor in patients with head and neck cancers, including NPC, and our previous large-scale prospective trial confirmed this finding (26, 27). In the present study, we demonstrated that low SMM impairs health-related QOL in NPC patients receiving CCRT. It is valuable to have a comprehensive understanding of the frequency and effects of low SMM in this population to: (1) identify characteristics of patients at risk of developing low SMM; (2) determine how low SMM can impact health-related outcomes; and (3) provide additional nutritional and functional assistance to support this population. By showing the negative effect of low SMM on patient QOL, our results provide strong evidence for supportive interventions. Rehabilitation and nutrition experts should participate in the multidisciplinary management of cancer patients in order to identify and treat patients with low SMM earlier in the course of cancer treatment. Such interventions may allow clinicians to better manage their patients, improve QOL, and even improve prognosis.

In our study, older patients were more likely to develop low SMM, consistent with previous publications (28, 29). Studies suggest that the decreased SMM and changes in muscle density observed in older patients may be attributable to neurological, nutritional, hormonal, metabolic, and physical activity-related factors (30, 31). Patient mobility and functional dependence can be affected by these changes in muscle, leading to disability and falls (32). These results indicate that for older cancer patients, screening and assessing SMM and nutritional and functional status may need to be early performed during the course of treatment. We also demonstrated that patients with lower PNI were more likely to have low SMM. PNI is a reflect factor for nutritional and immune status of cancer patients, which is a meaningful prognostic factor in NPC patients (20, 33). A possible reason for the above finding may be that stronger anticancer T-cell immunity may improve muscle quality and function through interaction with immune cells (34), it is more likely that a good muscle mass allows a better immune response, being a reserve for the production of acute-phase proteins. These results indicate that oncologists should screen and assess older cancer patients for sarcopenia and nutritional and functional status early in the course of treatment. Consistent with previous studies, we also found that male patients were more likely to develop low SMM (28, 35), which may be attributable to differences in the patterns of muscle loss between men and women. Moreover, the gender-specific cutoffs for low SMM, though adopted from consensus guidelines, may differ in specific populations. Overall, our results may help identify individuals who are at a high risk for developing low SMM, so that they may receive tailored interventions that meet their needs.

We are the first to report that low SMM, as evaluated using routine CT-sim images, impairs the health-related QOL in NPC patients receiving CCRT. Some researchers believe that low SMM-related physical decline has a negative impact on the QOL of cancer patients by reducing their functional ability (14). The association of pain symptoms with dysphagia and reduced physical activity may be a new explanation for the link between low SMM and pain in cancer patients. Importantly, our results will provide evidence for future efforts to evaluate and treat low SMM in the early stages of cancer treatment, which may improve the prognosis of patients with CCRT-related adverse events.

There are several limitations to this study. First, this study enrolled a small number of patients limited racial diversity from a single cancer center in an area where NPC is endemic. Thus, our results may not be generalizable, multiregional studies with larger samples are needed to confirm our results. Second, we enrolled patients from a prospective clinical trial; these patients may have different characteristics from those excluded from the prospective trial. Third, due to the lack of longitudinal CT scans, we could not determine changes in muscle mass throughout the course of cancer (patients may develop low SMM during or after cancer treatment) and their further effects on patient QOL. Finally, because our study was cross-sectional, we assessed the relationship between low SMM and patient QOL only over a short time; further research is needed to clarify the precise nature of this relationship.

## Conclusions

We have demonstrated that locoregionally advanced NPC patients receiving CCRT have a high incidence rate of low SMM as evaluated using routine head and neck CT simulation scans. Patients with low SMM experience worse QOL and higher pain scores, which highlights the urgency of nutritional and functional interventions to address improve SMM early during the course of treatment. Further researches should focus on when and how to evaluate and improve SMM to enhance the health-related QOL and pain control in NPC patients receiving CCRT.

## Data Availability Statement

The datasets generated for this study are available on request to the corresponding author. The data and materials of this study will be included at RDD (http://www.researchdata.org.cn/).

## Ethics Statement

The studies involving human participants were reviewed and approved by Institutional Review Board of Sun Yat-sen University Cancer Center. The patients/participants provided their written informed consent to participate in this study.

## Author Contributions

LG and H-XL: conceptualization, writing (review and editing), supervision, and project administration. XH, J-FL, and SL: methodology, formal analysis, and writing (original draft preparation). JZ, Z-QL, and XH: software. XH, H-YH, and W-WZ: validation. H-YH, XH, WW, SL, and J-FL: investigation. LG, H-XL, and W-WZ: resources and funding acquisition. XH and H-XL: data curation. W-WZ and H-YH: visualization.

### Conflict of Interest

The authors declare that the research was conducted in the absence of any commercial or financial relationships that could be construed as a potential conflict of interest.
